# Wearable Motion Capture Devices for the Prevention of Work-Related Musculoskeletal Disorders in Ergonomics—An Overview of Current Applications, Challenges, and Future Opportunities

**DOI:** 10.3390/s23094259

**Published:** 2023-04-25

**Authors:** Carl Mikael Lind, Farhad Abtahi, Mikael Forsman

**Affiliations:** 1 IMM Institute of Environmental Medicine, Karolinska Institutet, 171 77 Stockholm, Sweden; miforsm@kth.se; 2 Division of Ergonomics, School of Engineering Sciences in Chemistry, Biotechnology and Health, KTH Royal Institute of Technology, 141 57 Huddinge, Sweden; sabt@kth.se; 3 Department of Clinical Science, Intervention and Technology, Karolinska Institutet, 171 77 Stockholm, Sweden; 4 Department of Clinical Physiology, Karolinska University Hospital, 141 86 Huddinge, Sweden; 5 Centre for Occupational and Environmental Medicine, Stockholm County Council, 113 65 Stockholm, Sweden

**Keywords:** technical measurements, technical measurement instruments, upper limb posture, inertial measurement units, work technique training, ambulatory sensor systems, vibrotactile feedback, biomechanical exposure, biomechanical risk assessment, physical workload

## Abstract

Work-related musculoskeletal disorders (WMSDs) are a major contributor to disability worldwide and substantial societal costs. The use of wearable motion capture instruments has a role in preventing WMSDs by contributing to improvements in exposure and risk assessment and potentially improved effectiveness in work technique training. Given the versatile potential for wearables, this article aims to provide an overview of their application related to the prevention of WMSDs of the trunk and upper limbs and discusses challenges for the technology to support prevention measures and future opportunities, including future research needs. The relevant literature was identified from a screening of recent systematic literature reviews and overviews, and more recent studies were identified by a literature search using the Web of Science platform. Wearable technology enables continuous measurements of multiple body segments of superior accuracy and precision compared to observational tools. The technology also enables real-time visualization of exposures, automatic analyses, and real-time feedback to the user. While miniaturization and improved usability and wearability can expand the use also to more occupational settings and increase use among occupational safety and health practitioners, several fundamental challenges remain to be resolved. The future opportunities of increased usage of wearable motion capture devices for the prevention of work-related musculoskeletal disorders may require more international collaborations for creating common standards for measurements, analyses, and exposure metrics, which can be related to epidemiologically based risk categories for work-related musculoskeletal disorders.

## 1. Introduction

The WHO has estimated that about 1.71 billion people worldwide have musculoskeletal (ill-health) conditions [1]. Musculoskeletal conditions are the major contributor to disability worldwide, with low back pain (LBP) being the single largest cause of disability in 160 countries [1]. The annual global burden of work-related LBP has been estimated to be about 22 million disability-adjusted life years [2]. Of the global compensation costs of occupational- and work-related accidents and diseases, musculoskeletal disorders (MSDs) account for about 40% [3]. The societal economic cost has been estimated to be 3.9% of the global gross domestic product and 3.3% of the gross domestic product in the European Union [4]. Clearly, work-related diseases and disorders greatly burden companies and societies while affecting individuals’ wellbeing.

### 1.1. Musculoskeletal Disorders and Risk Factors

MSDs is an umbrella term for injuries and illnesses of the locomotor apparatus (i.e., tendons, ligaments, joints, nerves, vessels, and other supporting structures involved in locomotion) [5,6,7]. This includes the upper and lower body. Work-related musculoskeletal disorders (WMSDs) are MSDs that may be caused, aggravated, accelerated, or exacerbated by interaction with known or unknown factors in the workplace [5,6,7]. In addition to pain, ache, or similar, MSDs and WMSDs may impair work capacity, increase the risk of sickness and absence from work, and lead to premature exit from the labor market [8,9].

MSDs (including WMSDs) have a multifactorial origin and comprise physical and psychosocial risk factors, as well as individual factors (e.g., a previous history of illnesses, lower tolerance, and physical capacity, etc., and are sometimes associated with age, sex, and job tenure) [6,10,11,12,13]. Major occupational physical WMSD risk factors include manual handling (e.g., lifting, carrying, and pushing) [6,11,14,15,16,17,18,19,20,21,22,23], non-neutral postures (e.g., bending and twisting) [6,11,14,15,16,21,22,23,24,25,26], repetitive movements [6,11,13,14,27,28,29,30], hand-arm vibrations [6,11,14,27,31,32], and whole-body vibrations [6,11,33,34]. Generally, exposure to these factors does not pose an elevated risk if the intensity does not exceed the individuals’ tolerance levels and sufficient recovery is provided. However, many occupations and work tasks induce prolonged exposures and/or high-intensity levels. For example, according to self-reported data in the European Working Conditions Survey [35], about 70% of the employees in the European Union have work that comprises tiring or painful postures at least a quarter of the work time, and 13% have such work more than three-quarters of the work time. About the same percentage of employees (i.e., about 70%) have work that comprises repetitive hand or arm movements at least a quarter of the worktime, and 31% for more than three-quarters of the worktime. The development of MSDs is commonly a gradual process involving long latency periods and may initially manifest as fatigue or discomfort [6]. Without sufficient time for restorative repair (healing) [36], this can develop into more severe and persistent outcomes, such as disabling MSDs [7,37]. Due to this time lag [38], surveillance of early signs of MSD-related symptoms and exposure assessments are needed so that hazardous exposures can be identified at an early stage. From this, to prevent WMSDs, risk-reducing measures can be prioritized and implemented using the information to eliminate or reduce adverse exposures [39,40]. In such a way, the management of work environment risks can be structured proactively rather than taking action when symptoms of MSDs have emerged [39,41].

### 1.2. Obligations for the Management of Musculoskeletal Disorders at Work

Employers are generally required to take the measures necessary to secure workers’ safety and health protection, including preventing occupational risks and evaluating the risks that cannot be avoided [42]. Fundamental parts of risk management [43] include hazard identification, risk analysis, and risk assessment. With regard to the management of MSD risks, the risk assessment of biomechanical exposures is often performed by an expert, such as an occupational safety and health (OSH) practitioner (including occupational health and safety practitioners and ergonomists) [41,44,45]. It may include a combination of approaches to collect information, such as self-reports, observational tools, and technical measurements [46]. Traditionally, OSH practitioners have mainly relied on employees’ self-reports and observational-based tools to assess postures and movements of the trunk and upper extremities. In contrast, wearable motion capture instruments, such as accelerometers and inertial measurement units (IMUs), have been used less frequently. While self-reports and observational-based tools are versatile and can collect information about exposures retrospectively [47], these methodologies can usually not collect exposure measurements on a continuous scale. Instead, the exposure measurements are often of gross categories, often dichotomized [48,49], and often lack precision and accuracy for exposure measurements of the trunk and, especially, of the upper extremities [50,51,52,53,54]. Observation methods have been reported to have unsatisfactory inter- and intra-observer reliability [50,53]. The low use of technical measurement instruments in the past may at least partly be related to the high cost and complexity of the instruments, maintenance needs, low ease-of-use, being intrusive, insufficient robustness, and limited application (such as on a single joint). Some technical measurement instruments are restricted to stationary tasks due to wires connected to a computer. They utilize reflective markers requiring specific light conditions and are restricted in space (i.e., non-ambulatory). Additionally, while there are quantitative regulatory action and limit values [55,56] for noise and vibration exposures, no corresponding quantitative action and limit values exist for postures and movements of the body [57,58].

### 1.3. Wearable Technology

Wearable technologies, such as devices used for monitoring physical activity or motion of single body segments, have emerged by including sensing, processing, storage, and communication capabilities. A third feature includes usability and comfort. Based on key features of wearables recognized from various definitions, we have proposed a definition for wearable devices, presented in the last row of Table 1.

This definition sets a framework for identifying and categorizing wearable devices used in the prevention of WMSDs. In this article, the term wearable device comprises electronic or mechanical systems that perform a specific task or action. Hence, a sensor is a device that detects a specific type of physical input, while an instrument is a device that measures or manipulates variables, such as acceleration and magnetic field. A technical system is a set of components, e.g., a device/instrument that interacts toward a specific goal. In this context, wearables can be referred to as both devices or systems which comprise one or more instruments, and eventually, each instrument could include several sensors. The applications of wearable technologies have increased and are broadened into new areas, often in combination with machine learning [65], and most often they add value in relation to traditional non-sensor applications; see, e.g., [66].

### 1.4. Aim

Given the versatile potential for the use of wearables in the prevention of WMSDs of the trunk and upper extremities, this article aims to provide an overview of current applications and discuss challenges for the technology to support prevention measures and future opportunities, including important future research areas. The overview covers wearable motion capture instruments and systems targeting postures and movements of the trunk, arms, and wrists in mainly real occupational settings comprising an adult working population. This means that other marker-based and markerless motion capture systems that are restricted to stationary tasks and laboratory environments are excluded from this overview. Hence, an overview of, e.g., optical motion capture systems [67,68] or force plates [69] are outside the scope of this overview, along with other wearable sensorized instruments (since the overview is focused on postures and movements), such as surface electromyography (sEMG). Studies targeting sports or rehabilitation are also outside the scope of this overview. Instead, the reader is referred to other sources (e.g., [69,70,71,72,73,74,75,76,77,78,79]).

In this article, the term wearable motion capture instrument is used when referring to a single sensor unit or sensor assembly based on, e.g., a triaxial accelerometer or an IMU that can be used to obtain kinematic measures. The term wearable motion capture system is used for an assembly of single or multiple sensors connected to an external receiver that processes, records kinematic measures, and/or analyses the data.

## 2. Materials and Methods

Based on the broad focus of this overview, a narrative review methodology was applied. This means that we did not attempt to identify and include all relevant literature, but a sufficient number was used to identify some key aspects related to the use of wearables in the prevention of WMSDs. Only peer-reviewed literature was considered for inclusion. The literature was identified using a multi-faceted approach that involved screening recent literature reviews and overviews to identify literature from their reference lists, and from the reference lists of the identified articles (i.e., “snowballing”) [80,81]. Additional articles were identified by searches in the Web of Science platform (Clarivate Analytics PLC), and from the authors’ own digital libraries.

### 2.1. Wearable Motion Capture Instruments

For this narrative review, peer-reviewed literature published from the year 2001 to 2022 was included (see Section 3.1). Inclusion criteria were original studies written in English that were carried out in real occupational settings (i.e., not simulated work) and included an adult working population. For inclusion, the studies had to measure kinematic exposures (postures and/or movements) of the arm, trunk, and wrist using ambulatory motion capture instruments such as triaxial accelerometers, IMUs, and electrogoniometers, or similar. Since it can be challenging to obtain search terms with a high degree of sensitivity and specificity to identify field studies, two recently published literature overviews in 2021 [82] and 2022 [83] were used as a basis. From these two overviews, 57 studies were identified as fulfilling the inclusion criteria. To identify additional studies, the reference lists of these 57 studies were scanned for additional peer-reviewed articles. This was complemented by scanning for relevant literature from the authors’ own digital libraries. To identify a greater number of more recent studies (i.e., from 2020 to 2022), a literature search of peer-reviewed articles published from 2020 to 2022 in Web of Science was performed using the following search terms: “posture” OR “movements” OR “kinematics” AND “IMU” OR “inertial measurement unit” OR “accelerometer” AND “ergonomics”. Combining the articles from the authors’ digital libraries with the literature search resulted in an additional 29 peer-reviewed articles, resulting in a total of 86 included articles. From the 86 studies, the following information was obtained: (a) the country where the study was conducted; (b) the occupation or work; (c) the type of kinematics data recorded (i.e., postures and movements); (d) the targeted upper body segment(s) (arm, trunk, or wrist); (e) the type of wearable motion capture instruments that was used; and (f) the type of arm movement velocity reported (since not only one type is reported).

### 2.2. Ambulatory Motion Capture Systems

This search focused on identifying ambulatory motion capture systems, including a structure for automatically analyzing and categorizing the kinematics data designed to support exposure assessment or risk assessment of real work (see Section 3.2.3). Inclusion criteria were peer-reviewed literature (including conference papers) written in English published from the year 2001 to 2022 on an adult population. To identify peer-reviewed literature published until 2020, four recent systematic literature reviews published in the years 2018–2022 were screened (see Table 2).

From this, 17 peer-reviewed articles were identified that fulfilled the inclusion criteria. To identify studies until 2022, a recent overview of an original study was screened, resulting in four additional studies. To identify more recent studies, a literature search was conducted using Web of Science for peer-reviewed articles published from 2010 to 2022 using the following search terms: “posture” OR “movements” OR “kinematics” AND “IMU” OR “inertial measurement unit” OR “accelerometer” AND “ergonomics” AND “risk assessment”. This resulted in seven additional studies fulfilling the inclusion criteria, resulting in a total of twenty-eight included articles. From the 28 studies, the following information was obtained: the type and timing of the feedback provided; the type of exposure criteria (i.e., posture thresholds or risk assessment tools); the targeted body segment; and the type and location of the wearable motion capture instrument(s).

## 3. Application of Wearable Motion Capture Instruments and Systems for the Prevention of Work-Related Musculoskeletal Disorders

Wearable motion capture instruments and systems have multiple potential uses in risk management [43] for the prevention of WMSDs This includes exposure measurements (i.e., kinematic measures such as postures and movements of the trunk, arms, and wrists) that can be used as an input for hazard identification, risk analysis, and risk assessment. As shown in the research literature (e.g., [50]), motion capture instruments and systems generally have superior accuracy and precision of kinematic measures, as well as potentially lowering the associated costs in comparison to visual observations or analyses of video recordings (at least for large data samples when also considering the precision of the measurement [113]). Another use of motion capture instruments and systems includes risk assessment, i.e., in addition to providing kinematics measures as an input to the risk assessment, several motion capture systems have integrated one or several risk assessment tools (see Section 3.2). This can be used as a decision base to support assessing if the risk (or hazard) is acceptable or not, i.e., whether risk-reducing measures are needed or not [39,114]. The risk assessment can also support prioritizing risk-reducing measures to target the most severe risks and provide in-depth information on the underlying parameters affecting the risk to tailor efficient risk-reducing measures [39,40,114]. Risk-reducing measures should typically follow the so-called hierarchy of controls (see Section 3.3), for which both the exposure measurement and risk assessment provide important information. One important risk-reduction measure is to ensure that workers exposed to hazards have adequate training to mitigate the risks, e.g., work technique training for those performing hazardous manual handling. Some motion capture systems include a solution that automatically can generate feedback to the user (based on kinematic measures and automized risk assessment) to reduce adverse postural exposures by improving the work technique [85]. The following section gives examples of current applications and challenges in these areas, i.e., exposure measurements, risk assessments, and risk-reduction measures (feedback for work technique training).

### 3.1. Exposure Measurements

#### 3.1.1. Ambulatory Motion Capture Instruments and Systems

**Figure 1:**
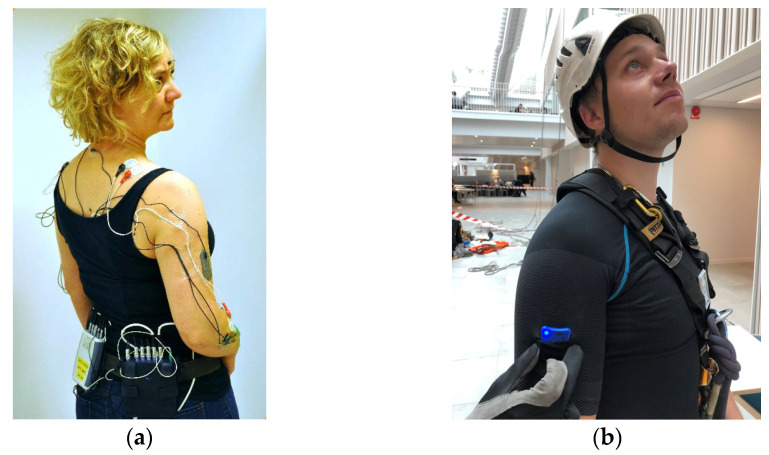
Commonly used electrical instruments for measurements of postures and movement of the arms and trunk. (**a**) Triaxial accelerometers connected to a data logger (from Arvidsson et al. [115], with permission) and (**b**) an inertial measurement unit connected to a smartphone.

Postures and movements of the trunk, arms, and wrists can be monitored in occupational settings with motion-tracking instruments placed on the targeted body segment. Among electrical motion capture instruments (Figure 1), triaxial accelerometers have been commonly used for measurements of postures and movement of the arms and trunk, while twin-axis electrical goniometers are often used for kinematic measurements of the wrist.

As shown in Table 3, IMUs are increasingly being used for kinematic measurements of the arms and trunk [69,116], while twin-axis goniometers (electrogoniometers) still seem to dominate kinematics measurements of the wrist [46,117].

Trunk movements have usually been reported as inclination velocity in the forward (sagittal) [118] or sideways (lateral) direction [119]. For the recording of arm movements, however, two fundamentally different types of velocities are currently being reported: inclination velocity and generalized velocity [82,83,120]. A third, i.e., gyroscope vector magnitude, has recently been proposed as an alternative [58]. As shown by Fan et al. [82], the differences due to sensor type (i.e., accelerometer or IMUs) and angular velocity computational method (i.e., inclination velocity or generalized velocity) can render large differences, e.g., 4.5 times higher estimated angular arm velocity for generalized velocities from accelerometers when compared to inclination velocity from IMUs. This creates obvious challenges when exposure measurements obtained using different sensor types or angular velocity computational methods are to be merged or compared [120]. One possible solution for this is to re-calculate the data, i.e., using conversion equations developed by Forsman et al. [83]. By applying the conversion equations, exposure measurements obtained using different sensor types, angular velocity computational methods, and sampling frequencies can be harmonized. The generalizability of such conversion equations to multiple occupational groups is yet to be tested; hence, more research is needed.

**Table 3:** The table shows peer-reviewed articles of studies from the year 2001 to 2022 collecting kinematics data of the arm, trunk, and wrist performed in the field of real work. The table shows the country where the study was conducted (Origin) and which occupation or work was monitored (Occup). For each body segment, the table shows which sensor type was used and whether postures and movement/angular velocity were recorded (indicated by “X” if they were monitored). The motion capture instruments used include triaxial accelerometers (acc), inertial measurement units (IMUs), potentiometers (pot), and electrogoniometers (gon). Since different angular velocity types were reported for the arm, the type of arm angular velocity is shown, i.e., generalized arm velocity (Gen) or inclination arm velocity (Inc).

Ref.	Year	Origin	Occup.*	Arm			Trunk			Wrist		
				Sens.	Post.	Vel.	Sens.	Post.	Vel.	Sens.	Post.	Vel.
[121]	2001	DK	PP	acc	X	Gen	acc	X	X	gon	X	X
[122]	2002	SE	CAD	acc	X	Gen	acc	X	X	gon	X	X
[123]	2002	SE	IMF	acc	X	Gen	acc	X	X	gon	X	X
[124]	2004	US	CS	-	-	-	gon	X	X	-	-	-
[125]	2005	SE	CD	acc	X	Gen	acc	X	X	gon	X	X
[126]	2006	SE	IMF	acc	X	Gen	-	-	-	gon	X	X
[127]	2006	SE	ATC	acc	X	Gen	acc	X	X	gon	X	X
[128]	2006	SE	ATC	acc	X	Gen	-	-	-	gon	X	X
[129]	2007	SE	Cl	acc	X	Gen	-	-	-	gon	X	X
[130]	2007	DE	Nu	-	-	-	IMU ^1^	X	-	-	-	-
[131]	2008	DE	OD	-	-	-	IMU ^1^	X	-	-	-	-
[132]	2008	NO	HD	acc	X	Gen	-	-	-	-	-	-
[133]	2008	SE	IMF	acc	X	Gen	-	-	-	gon	X	X
[134]	2008	US	IMF	acc	X	-	-	-	-	gon	X	X
[135]	2009	SE	De	acc	X	Gen	-	-	-	-	-	-
[136]	2010	DE	OD	-	-	-	IMU ^1^	X	-	-	-	-
[137]	2010	US	CS	acc	X	Inc	-	-	-	-	-	-
[138]	2010	SE	HD	acc	X	Gen	-	-	-	-	-	-
[139]	2011	BR	CE	acc	X	Gen	-	-	-	-	-	-
[140]	2011	SE	De	acc	X	Gen	-	-	-	-	-	-
[141]	2011	US	HC	-	-	-	acc	X	-	-	-	-
[142]	2011	US	CS	-	-	-	acc	X	-	-	-	-
[143]	2012	US	Lo	-	-	-	gon	X	X	-	-	-
[144]	2012	NL	OW	acc	X	-	-	-	-	gon	X	X
[145]	2012	BR	EU	acc	X	-	-	-	-	-	-	-
[146]	2012	US	Da	acc	X	Inc	-	-	-	-	-	-
[147]	2012	SE	MC	acc	X	Gen	-	-	-	gon	X	X
[148]	2012	SE	De	acc	X	Gen	-	-	-	gon	X	X
[149]	2012	DE	Nu	-	-	-	IMU ^1^	X	-	-	-	-
[150]	2013	SE	De	acc	X	Gen	acc	X	X	-	-	-
[151]	2013	NO/BR	El	acc	X	-	-	-	-	-	-	-
[152]	2013	US	De, Co	acc	X	Inc	-	-	-	-	-	-
[153]	2014	US	Lo	-	-	-	gon	X	X	-	-	-
[154]	2014	AU	OW	pot	X	-	pot	X	-	-	-	-
[155]	2014	DK	HP	acc	X	Gen	-	-	-	gon	X	X
[156]	2014	DE	Nu	-	-	-	IMU ^1^	X	-	-	-	-
[157]	2015	NO	Var	acc	X	-	-	-	-	-	-	-
[119]	2016	SE	ABH	acc	X	Gen	acc	X	X	-	-	-
[158]	2016	SE	GS	acc	X	Gen	-	-	-	gon	X	X
[118]	2016	US	Nu	IMU	X	Inc	IMU	X	X	-	-	-
[159]	2016	US	Da	IMU	X	-	IMU	X	-	-	-	-
[160]	2016	DE	De	-	-	-	IMU + pot	X	-	-	-	-
[161]	2016	DE	OD	-	-	-	IMU	X	-	-	-	-
[162]	2017	DE	OD	-	-	-	IMU	X	-	-	-	-
[163]	2017	DE	Nu	-	-	-	IMU	X	-	-	-	-
[164]	2017	SE	Su	IMU	X	Inc	IMU	X	X	-	-	-
[96]	2017	FR	FC	IMU	X	-	IMU	X	-	gon	X	-
[165]	2017	US	Cl	-	-	-	acc	X	-	-	-	-
[166]	2017	FR	IMF	acc	X	-	acc	X	-	gon	X	-
[167]	2018	DK	Var	acc	X	-	-	-	-	-	-	-
[168]	2018	SE	SG	acc	X	Gen	-	-	-	gon	X	X
[169]	2018	SE	Cl	acc	X	Gen	-	-	-	-	-	-
[170]	2018	DE	De	-	-	-	IMU + pot	X	-	-	-	-
[171]	2018	US	Agr	IMU	X	Inc	IMU	X	X	-	-	-
[172]	2019	US	IMF	IMU	X	Inc	-	-	-	-	-	-
[173]	2019	BR	Agr	IMU	X	-	IMU	X	-	IMU	X	-
[174]	2019	SE	IMF	acc	X	Gen	acc	X	X	-	-	-
[175]	2019	DK	BC	acc	X	-	acc	X	-	-	-	-
[176]	2019	NO	HD	acc	X	-	-	-	-	-	-	-
[177]	2019	NO	CS, HC	acc	X	-	acc	X	-	-	-	-
[175]	2019	DK	BC	acc	X	-	acc	X	-	-	-	-
[178]	2019	US	AH	acc	X	-	acc	X	-	-	-	-
[179]	2020	DE	Ne	-	-	-	gon	X	-	-	-	-
[180]	2020	US	AH	acc	X	-	acc	X	-	-	-	-
[181]	2020	US	AH	acc	X ^4^	-	-	-	-	-	-	-
[182]	2020	FR	VA	acc	X	-	acc	X	-	-	-	-
[183]	2020	DK	CC	acc	X	-	acc	X	-	-	-	-
[184]	2020	IR	Ba	acc	-	Inc	-	-	-	-	-	-
[185]	2020	CA	Lo	IMU	X	-	IMU	X	-	-	-	-
[186]	2020	US	Agr	IMU	X	Inc	IMU	X	X	IMU	-	X
[187]	2020	CA	Fa	-	-	-	IMU	X	X	-	-	-
[188]	2020	IT	Lo	-	-	-	IMU	X	-	-	-	-
[189]	2021	SE	OD	acc	X	Gen	acc	X	X	gon	X	X
[190]	2021	DE	Lo	-	-	-	IMU	X	-	-	-	-
[191]	2021	US	IMF	IMU	X	Inc	IMU	X	X	-	-	-
[192]	2021	US	IMF	-	-	-	IMU	X	-	-	-	-
[82]	2021	SE	Lo	IMU ^2^	X	Inc + Gen	-	-	-	-	-	-
[110]	2021	US	CS	IMU	X	-	IMU	X	-	-	-	-
[26]	2022	DK	Var	acc	X	-	-	-	-	-	-	-
[25]	2022	DK	Var	-	-	-	acc	X	-	-	-	-
[193]	2022	US	Fi	IMU	X	-	IMU	X	-	-	-	-
[194]	2022	PT	VA	IMU	X	-	IMU	X	-	IMU	X	-
[195]	2022	SE	OD	acc	X	Gen	IMU	X	X	-	-	-
[196]	2022	SE	Su	IMU	X	Gen	IMU	X	X	-	-	-
[83]	2022	SE	Lo	IMU	X	Inc + Gen	-	-	-	-	-	-
[112]	2022	CL	MC	-	-	-	-	-	-	IMU	X	-
[85]	2023 ^3^	BE	Lo	-	-	-	IMU	X	-	-	-	-

Notes: * abbreviation for occupations: ABH (aircraft baggage handlers); Agr (agricultural work); AH (apple harvesting); ATC (air traffic controllers); BC (blue-collar sector occupations); CAD (computer-aided designer); CC (childcare); CD (car disassembly); Ba (bakers); CE (construction electricians); Cl (cleaners); Co (computer workers); CS (construction workers); Da (dairy workers); De (dentistry); El (electricians); EU (electric utility workers); Fa (farming); Fi (fishing); FC (filter cleaning); GS (grocery store workers); HC (healthcare); HD (hairdressers); HP (house painters); IMF (industrial manufacturing occupations); Lo (logistics); MC (meat cutters); Ne (neurology); Nu (nursing); OW (office workers); OD (occupational drivers); PP (poultry processing); SG (sonographs); Su (surgeons); VA (vehicle assembly); Var (various occupations); ^1^ this version of the CUELA system seems to contain IMUs but is based on the information presented; ^2^ IMUs were additionally used to simulate the use of accelerometers; ^3^ published as “in-press” in 2022; ^4^ postures are reported as repetition of movements.

The location and attachment of the motion capture instruments vary, and this can also alter the estimated exposure [197]. For example, for recordings of arm postures and movement, the wearable motion capture instruments are often placed in alignment with the axis of the humerus and below the insertion of the deltoideus muscle [82], but alternative placement occurs further distal, approaching the elbow joint [87] or proximal, i.e., in top of the deltoideus muscle [119]. The latter position can render differences of up to about 5–7° at the group level [197].

The attachment of the instruments also varies in field studies, i.e., from being attached directly to the skin using double-sided adhesive tape [82], using a strap [87], being placed in a pocket of the clothes [104,105], or mounted on a plate that is attached to the skin. To what extent these different attachments affect the measurements is not fully understood, but it has been suggested that this potentially can contribute to disparate measurements [198] attributed to soft tissue artifacts [199].

Other systems integrate textile-based (stretch) sensors into the clothes [200]. The textile-based systems utilize actuating fibers, yarns, or fabric to sense external mechanical (stretch) stimuli. These systems can currently be used for classifying (gross) physical activities such as walking, carrying, lifting, pushing, and pulling [200,201].

Many wearable motion capture instruments allow capturing data continuously over multiple workdays or even up to 2–3 weeks, depending on the sample rate (e.g., Axivity AX3, ActiGraph GT3X). Due to the small size of many wearable motion capture instruments, the devices can be worn for several workdays without having to be taken off during sleep or other activities, which reduces the need to have them attached before each workday [202]. However, for some systems where the sensors transmit real-time data, the maximum data collection period is often significantly shorter because of battery capacity, which is about 4–6 h or less [104]. This is a current challenge when the aim is to provide feedback during whole workdays without changing or re-charging the instruments during the workday.

As can be seen in Table 3, we have not identified any studies where IMUs have been used to measure wrist angles in real occupational settings (only velocity [186]). There are, however, a few laboratory-based validation studies that have shown high correlations (>0.9) between wrist angles measured with IMUs and marker-based optical motion capture systems in simulated manual material handling tasks [203] and in simulated swimming [204]. However, these two studies were performed without ferromagnetic materials close to the subjects, and the influence of ferromagnetic objects in a real workplace remains unclear [116,205].

Recently, IMUs have been introduced and evaluated against twin-axis electrical goniometers for recording wrist velocities in simulated work showing promising results with close correspondence both for standardized flexion/extension movements (correlation close to one and less than 10% difference in the 50th and 90th percentiles, and in three simulated work tasks with mean differences below 10%) [117]. Only the gyroscope data was used in the velocity computations, and there was no magnetic field dependence. The approach has recently been used within a smartphone app, which has been shown to have high usability in field measurements [206].

**Figure 2:**
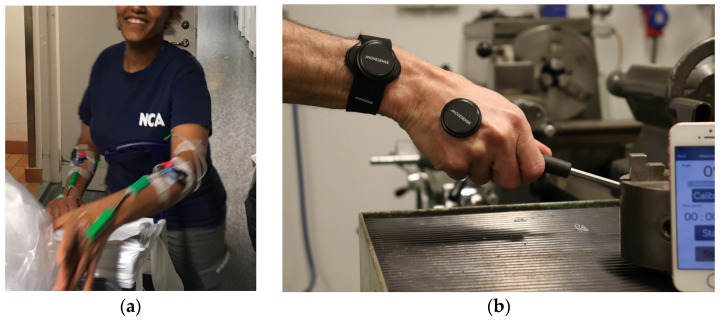
An example of (**a**) electrogoniometers and (**b**) an inertial measurement unit.

Although we have identified one study where IMUs have been used for wrist velocity measurements [186], wearable motion capture of the wrist has been dominated in the last three decades by electrogoniometers (Figure 2a). While they are reliable and sufficiently accurate, they are more expensive than the new IMUs (Figure 2b), and the mechanical robustness is usually low. In occupational measurements, they typically break within a week of usage.

In addition to those systems targeting single body segments or joints, or mainly the upper body, other systems target the whole body using up to 17 IMUs. Such a system (Xsens MVN, Enschede, The Netherlands) has been validated for many joint angles [205] and for the estimation of L5/S1 moment during manual material handling tasks [207] in a laboratory environment without ferrimagnetic materials in the proximity of the subjects. The full-body system (Xsens MVN) has also been tested in a workplace [185], showing acceptable feasibility, but it was concluded that visual verification remains important for scientific rigor because of possible errors from magnetic field disturbances. This and similar comprehensive systems are still expensive and complex to use.

#### 3.1.2. Exposure Metrics and the Need for Harmonization

Currently, no single harmonized form of metrics reporting exists for either postures or velocities. Instead, different metrics are reported, as shown in Table 4. While some metrics tend to be more frequently reported (e.g., the 10th, 50th, and 90th percentiles), others are less frequent (e.g., the 1st percentile and the 5th–95th percentile range). The mean and standard deviation (SD) are usually reported, but a few studies instead report the median, range, and confidence intervals [85,125]. Additionally, a few studies report the within- and between-subject variability, in addition to the group mean values (e.g., [119,174]). A few have also reported a combination of postures and angular movement velocities to create indices aiming to capture, e.g., rest time, such as a combination of a “neutral” posture (e.g., <20°) in combination with a “slow” velocity (e.g., <5°/s). Commonly, many of the earlier studies shown in Table 4, have reported exposure of one arm (typically the dominant arm or the right arm), while many of the more recent studies report exposures of both arms (e.g., [118,119,174,191]).

The general lack of congruence of sensor position of the body, sensor type, computational method, and reported metrics all complicate comparisons of data between studies (between-studies comparison), which makes it challenging to compare exposure levels between different working populations as well as similar working populations across regions of the world. Hence, there exists a potential for further harmonization in this area, although previous efforts at such harmonization exist [208,209].

### 3.2. Risk Assessment

Risk assessment is a process within risk management comprising the identification of risks or hazards, estimating the risk level (probability and severity of the harm), and evaluating if the risk is acceptable or not (i.e., if risk-reducing measures are needed) [210]. Risk evaluation also includes the evaluation of implemented risk-reducing measures to ensure they have the intended effect of reducing the risk to an acceptable level without introducing new (unacceptable) risks. A few wearable motion capture systems include, in addition to exposure measurement, a system that facilitates risk estimation of WMSDs and/or risk evaluation to facilitate priorities for risk-reducing measures [39] (see Section 3.3).

**Table 4:** Showing the range of metrics (such as percentiles of the proportion of time) for kinematics data reported in a selection of the articles presented in Table 3. The examples below show arm exposures of the dominant or right arm in these articles, where “X” indicates the metrics used in each of the articles.

Ref.	[121]	[125]	[128]	[129]	[138]	[147]	[146]	[150]	[155]	[118]	[158]	[119]	[171]	[169]	[167]	[171]	[177]
Year	2001	2005	2006	2007	2010	2012	2012	2013	2014	2016	2016	2016	2018	2018	2018	2018	2019
Posture/Angle																	
Percentiles																	
1st														X			
10th	X	X	X	X	X		X			X		X	X	X			
50th	X	X	X	X	X	X	X	X		X		X	X	X	X		
90th	X	X	X	X	X	X	X			X		X	X	X	X		
99th		X			X	X			X		X	X		X	X		
90th–10th		X			X		X	X		X		X					
95th–5th			X														
Proportion of time																	
<15°																	
<20°					X		X			X		X					
<30°													X				
* >30°														X			X
* >45°							X			X							
* >60°		X			X		X			X		X	X	X	X	X	X
* >90°									X			X		X	X	X	
Movement (angular velocity)																	
Percentiles																	
10th		X			X		X			X		X	X				
50th	X	X	X		X	X	X	X	X	X	X	X	X				
90th	X	X	X		X		X			X		X	X				
99th		X			X							X					
90th–10th							X	X		X				X			
Proportion of time																	
<5°/s		X					X			X			X				
* >90°/s		X			X		X			X	X	X	X				
Combinations (posture and angular velocity)																	
% <15° and <5°/s																	
% <20° and <5°/s		X			X		X					X					
% <30° and <5°/s													X				
% <15° ≥3 s																	
% <20° ≥3 s							X			X							
% <30° ≥3 s													X				
% <5°/s ≥3 s					X		X			X		X	X				
% <20° & <5°/s ≥3 s							X			X							

Notes: * “>” or “≥”; “% <15° and <5°/s” means that the proportion of time where the arm is held is less than 15° from the vertical line of gravity, while (simulations) the angular velocity is less than 5°/s; “% <15° ≥3 s” means that the proportion of time where the arm is held is less than 15° from the vertical line of gravity, which is continuous for at least 3 s.

#### 3.2.1. Quantitative Metrics for Risk Assessment

Due to the often-superior accuracy and precision of wearable motion capture instruments, when compared to observational tools, wearable motion capture instruments can be a useful complement in risk assessments of WMSDs. However, the exposure measurements need to be translated to relevant metrics, such as whether the risk for WMSDs (e.g., back pain [211,212]) is acceptable. Despite this need, there is generally a lack of quantitative metrics derived from longitudinal epidemiological studies with exposure measurements from wearable motion capture instruments associated with WMSDs. This scarcity can obstruct the interpretation of quantitative measurements to meaningful guidance for decisions. Recently, however, Arvidsson et al. [58] presented a proposal comprising 13 quantitative threshold levels that can be used as indicators of hazardous exposure for WMSDs of the upper extremities, called action levels (Table 5). The action levels are based on a large sample of cross-sectional data and comprise over 1000 recordings of employees from about 60 different occupations and include physical examinations of WMSD symptoms. The action levels are intended for monitoring exposures using instruments such as accelerometers, IMUs, electrogoniometers, and sEMG and comprise threshold values for posture and movements of the arm, wrist, and head (inclination) posture), as well as muscle activity. If any of the action levels are exceeded, this indicates an increased risk for WMDs and should initiate measures to reduce the exposure below the action level.

Another example of systematically collected data is the database for the Lumbar Motion Monitor (LMM) [215,216,217], which uses three-dimensional (i.e., sagittal, lateral, and twisting plane) trunk postures and movements as the input to connect the kinematics data to a risk category related to LBP or low back disorders). A third example is the database by Gupta et al. [25,26], which targets both trunk postures and arm postures. The authors found dose–response associations between time of exposure over a limit (i.e., arm elevation above 30°, 60°, and 90°; forward trunk bending more than 30° and 60°) and prospective register-based long-term sickness absence during the four years following the measurements.

#### 3.2.2. Risk Assessment Using Observational Tools

An alternative to the objective quantitative metrics presented above is to use some of the many observational tools targeting the assessment of MSDs [7,50,76,218] (see Section 3.2.2 for examples of integration with wearable motion capture systems). This section will give a brief introduction to the use of these tools from the perspective of preventing WMSDs and some possible applications, as well as limitations for integration of the tools with wearable motion capture systems.

Currently, risk assessments of MSDs by OSH experts, such as occupational health services, are often performed with the support of observational tools. The scope and targeted application of each tool largely differ [7,50,114,219,220]. Typically, the tools target a limited number of body segments, exposures, or occupational tasks (see Table 6). For example, the Strain Index [221], the Revised Strain Index [222], the Distal Upper Extremity Tool [223], the Assessment of Repetitive Tasks (ART) tool [224], and the Hand Arm Risk Assessment method (HARM) tool [225] all target mainly repetitive tasks of the upper extremities, while some the other tools are mainly restricted to manual lifting and lowering tasks, such as the Revised NIOSH Lifting Equation [211] and the Lifting Fatigue Failure Tool [226]. Other tools, such as the Rapid Upper Limb Assessment (RULA) [227], the Rapid Entire Body Assessment (REBA) [228], and the Ovako Working posture Assessment System (OWAS) [229], target demanding postures and force exertion of the whole body. Additionally, the RAMP tool (Risk management Assessment tool for Manual handling Proactively) covers a broad range of body segments, exposure, and task, e.g., demanding postures, manual lifting, and manual pushing and pulling [51,52,114,230].

In addition to physical factors, such as postures and manual handling, a few of these tools also target mental/psychological/psychosocial factors [231], such as the ART tool and the RAMP Tool.

The use of each specific tool varies among countries [7,41,44,46,232,233,234,235], where the Revised NIOSH Lifting Equation, RULA, and the Psychophysical Material Handling Data [236,237] are among the most commonly used by occupational safety and health (OSH) experts (such as professional ergonomists) in USA, Canada, UK, Australia, and New Zealand [44,46] (68–91%). The two former tools are also commonly used by other OSH practitioners, e.g., the Revised NIOSH Lifting Equation and RULA were reported to be used, respectively, by 59% and 80% of Spanish-speaking OSH practitioners [234]. Examples of important qualities of observation tools include ease of use, added value for addressing relevant risks, ability to facilitate prioritization of measures, and being backed up by a relevant regulatory body [7,41,234].

Recent research has shown that wearable motion capture instruments, such as accelerometers and IMUs, are less frequently used among OSH experts when compared to observational tools. For example, even among highly skilled ergonomists in the USA, Canada, UK, Australia, and New Zealand (86% having at least a master’s degree and about 1/3 having a doctoral degree), fewer than 10% used (non-optical) motion capture instruments at least once every three months, and only about 5% at least once a month [46]. This can be compared to the Revised NIOSH Lifting Equation and the Psychophysical Material Handling Data, of which about 50% had used at least once every three months and about 30–35% at least once a month [46]. Other measurement instruments, such as push/pull force sensors (e.g., dynamometers/force gauges) and grip and pinch dynamometers, were used relatively more frequently, i.e., around 25–37% at least once every three months [46].

This, together with shown unsatisfactory inter- and intra-observer reliability of the observation methods [238], illustrate the potential for increased use of wearable motion capture instruments if they are perceived as feasible and usable and add extra value to the measurement. From this perspective, the precision and accuracy provided by wearable motion capture instruments (when compared to many observational tools) may be necessary to detect clinically relevant improvements following the implementation of risk-reducing measures [238], such as reduced time in trunk inclination [239], that sometimes may not be detected using observational tools. Additionally, when taking into account the precision of the measurement, wearable motion capture instruments can be more cost-efficient when compared to observational tools [113].

#### 3.2.3. Integration of Observational Risk Assessment Tools with Wearable Motion Capture Systems

Some wearable motion capture systems incorporate a structure for automatic connecting of the measurements to risk levels, often using observational risk assessment tools as a basis or predetermined threshold values (Table 7). The systems range from those focusing on a single joint (or body segment) using a single sensor to those covering multiple joints using, e.g., up to 17 motion capture instruments. A few of these systems also facilitate the information to be fed back to the user in real-time (concurrent feedback) or after completing a task (terminal feedback). As shown in Table 7, RULA is among the most commonly observational risk assessment tools to be integrated with wearable motion capture systems. However, most of the included studies use posture thresholds that are not directly related to thresholds derived from an observational risk assessment tool.

An advantage of integrating the observational risk assessment tools with the wearable motion capture systems is that this facilitates the quantitative measurements to be grouped into risk categories, sometimes taking into account complex combinations of postures, such as multiple body segments, and taking into account interaction effects [87]. On the other hand, connecting the measurements from the wearable motion capture systems to just a few gross categories lowers the granularity of the data.

As shown in Table 8, some of these observational tools (e.g., RULA, REBA, and OWAS) focus mainly on capturing snapshots of single hazardous events or a compilation of a few hazardous events, while other tools instead aim at the entire cumulative exposure of a work task or a job.

**Table 7:** Examples of wearable motion capture systems developed for assessing MSD risks related to postures and movements (see Table 2). Some of the systems can provide feedback directly to the wearer using different modalities: concurrent, visual, and auditory (see Section 3.3.2. for a description of different feedback characteristics).

Ref.	Year	Real-Time Processing	Feedback Timing	Feedback Modality	Exposure Criteria	Targeted Body Segment	Instrument	Instrument Location
[86] ^6^	2009	Yes	Concurrent	Visual and auditory	Posture thresholds	Neck	1 acc	Neck (C7 vertebrae)
[88]	2013	Yes	Concurrent	Vibrotactile	Posture thresholds	Trunk	1 strain gauge	Trunk (L3 and S2 vertebrae)
[87]	2013	Yes	Concurrent	Visual	RULA	Upper body	7 IMUs	Head Trunk (sternum/chest) Upper arm (bilateral) Wrist (bilateral) Sacrum
[92]	2014	n/a ^1^	Terminal ^2^	Visual	OWAS, OCRA index, RULA, and NIOSH lifting index	Whole body	17 IMUs	Head Trunk (upper and lower back) Shoulder blade Upper arm (bilateral) Forearm (bilateral) Wrist (bilateral) Upper leg (bilateral) Lower leg (bilateral) Feet (bilateral)
[89]	2014	Yes	Concurrent	Auditory	Posture thresholds	Trunk	1 accelerometer	Hipp
[90]	2014	-	Terminal	Visual	Posture thresholds	Neck and upper back	2 acc	Head (back) Trunk (upper back)
[91]	2014	Yes	-	Visual ^7^	RULA	Upper body	3 IMUs	Arm Wrist Hand
[93] ^6^	2015	Yes	Concurrent	Vibrotactile	Posture thresholds	Upper back	1 acc	Trunk (upper back)
[94]	2016	Yes	Terminal ^2^	Visual ^3^	Gross postures	Upper and lower body	7 IMUs	Trunk (back) Upper arm (bilateral) Forearm (bilateral) Shins (bilateral)
[95]	2016	Yes	-	-	RULA and Strain Index	Upper body	3 IMUs	Arm Wrist Hand
[98]	2017	Yes	-	Visual ^7^	ISO 11226:2000 [240]	Upper and lower body	8 IMUs	Trunk (lower and upper back) Upper arm (bilateral) Upper leg (bilateral) Lower leg (bilateral)
[97]	2017	Yes	Concurrent	Auditory	ISO 11226:2000 [240]	Upper body	2 IMUs	Trunk (upper back) Head (backside)
[96]	2017	-	-	-	RULA	Upper body	7 IMUs, 2 electrogoniometers	Head Trunk (chest) Upper arm (bilateral) Forearm (bilateral) Pelvis (sacrum) Wrist
[100]	2017	n/a ^1^	-	-	Posture thresholds (also time-dependent)	Whole body	4 IMUs ^4^, 1 potentiometer, 1 flex sensor ^4^	Trunk Arm ^4^ Forearm ^4^ Wrist ^4^ Thigh Calf
[99]	2018	Yes	Concurrent	Auditory	Posture thresholds	Trunk	2 acc	Trunk (upper and lower back)
[101]	2018	Yes	Concurrent	Vibrotactile	Posture thresholds	Trunk	1 acc	Trunk (center of left clavicle)
[102]	2019	Yes	Concurrent	Vibrotactile	Posture thresholds	Trunk	1 acc	Neck (posterior)
[103]	2019	Yes	Concurrent	Visual + auditory	Posture thresholds	Trunk	2 IMUs	Trunk (L1 and L5 vertebrae)
[109]	2020	Yes	Concurrent	Visual + vibrotactile	Posture thresholds (based on RULA and LUBA)	Neck, trunk, and arms	4 IMUs	Head (back) Trunk (T4 vertebrae) Upper arm (bilateral)
[106]	2020	Yes	-	-	RULA and REBA	Upper and lower body	17 IMUs	Forehead Trunk (2 front, 1 back) Upper arm (bilateral) Wrist (bilateral) Hand (bilateral) Pelvis/sacrum (front) Upper leg (bilateral) Lower leg (bilateral) Ankle (bilateral)
[105]	2020	Yes	Concurrent	Vibrotactile	Posture thresholds	Trunk and upper arm	2 IMUs	Trunk (T1–T2 vertebrae)Upper arm (dominant)
[104]	2020	Yes	Concurrent	Vibrotactile	Posture thresholds	Upper arm	1 IMU	Upper arm (dominant)
[107]	2020	Yes	Concurrent	Auditory	Posture thresholds	Trunk	1 acc	Hipp
[108]	2020	Yes	Concurrent	Auditory	Posture thresholds	Trunk	2 IMUs	Trunk (T0 vertebrae and sacrum)
[111]	2020	Yes	Concurrent	Auditory ^5^	Posture thresholds	Trunk	2 IMUs	Trunk: T0 vertebrae and sacrum
[110]	2021	Yes	Terminal	Visual	OWAS and MHT	Whole body	5 IMUs	Head Trunk (chest) Upper arm (dominant) Thigh (dominant) Calf (dominant)
[112]	2022	-	-	-	RULA	Wrist	1 IMU	Wrist
[85]	2023	Yes	Concurrent	Vibrotactile	Posture thresholds	Trunk	1 IMU	Trunk (T1–T2 vertebrae)

Notes: n/a (no answer); ^1^ possible, but insufficiently described for an independent assessment; ^2^ potentially also concurrent feedback but insufficiently described for an independent assessment; ^3^ potential for audio and vibration according to the authors themselves; ^4^ information cannot be identified if also bilateral; ^5^ potential for vibration according to the authors themselves; ^6^ not fully wearable; LUBA (postural loading on the upper body assessment) [241]; MHT (maximum holding times) [242]; ^7^ only feedback to the observer.

An advantage of these “snapshot” tools is that they usually comprise multiple categories for the classification of postures, while other observational tools that, to a greater extent, consider the cumulative exposure, such as RAMP, ART, and HARM, usually comprise fewer categories for the classification of posture angles. RULA, for example, includes five separate quantitative categories of the (upper) arm posture in the sagittal plane, whereas HARM comprises three categories (Table 9).

RULA has four neck flexion/extension categories in the sagittal plane (i.e., head sagittal inclination), while both RAMP and HARM have three categories (Table 10). However, RAMP and HARM combine this with a duration (3 categories in HARM and 5–7 categories in RAMP), while RULA does not (Table 11). Instead, RULA only gives an additional penalty (increased score) if the posture is held static for longer than 1 min without a break or repeated more than 4 times per min.

A disadvantage of the snapshot-based tools is the difficulty of summarizing the cumulative exposure of a full workday into a single risk score or risk category since the tools do not support this. This is contrary to some other tools, such as RAMP II and HARM, which support summarizing the exposure measurements of full workdays. However, the fewer categories (e.g., for the classification of postures) do, to a lesser extent, utilize the benefit of continuous exposure data provided by wearable motion capture instruments.

Another challenge is how to convert qualitative metrics to quantitative metrics. As an example, the ART tool comprises two qualitative categories for the (upper) arm posture: (elbow) kept close to the body or supported and (elbow) raised away from the body. This is also combined with a non-overlapping duration: part of the time (15–30%) and more than half of the time (>50%). Hence, to allow the integration of measurement from wearable motion capture instruments to tools such as ART, both the posture category and time category need conversion to quantitative overlapping categories. Similar challenges also exist for quantifying what constitutes a repetitive movement [244], which is used in many observational “risk assessment” tools.

These examples illustrate some of the current challenges when using observational risk assessment tools as a basis for measurements obtained by wearable motion capture instruments. Another challenge for utilizing current observational tools with wearable measurement systems is that most available observational tools’ quantitative or qualitative values (or categories) are mainly based on measurements originating from self-reports or observational tools [51]. As shown in several studies, both of these tools may lead to measurements that substantially under- or overestimate the time in specific posture angles (e.g., such as having the hands above shoulder height [54]) or the movement velocities when compared to measurements from a more valid source [121,245,246]. Therefore, caution is needed when interpreting exposure levels from these observational tools when measurements are derived from wearable measurement instruments.

### 3.3. Risk-Reducing Measures

**Figure 3:**
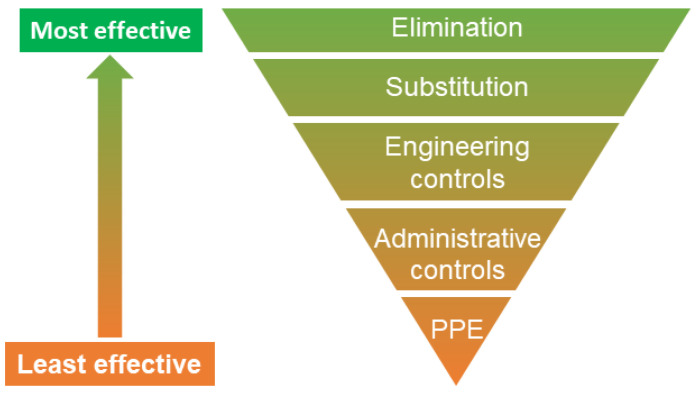
The hierarchy of controls displays the order in which hazards should be moved based on their general effectiveness. Based on a model from the National Institute for Occupational Safety and Health [219].

If manual handling is performed, employers in, for example, the European Union, are required to ensure that employees receive proper training on handling loads safely to avoid the risk of physical overload and MSDs [247]. In addition to using wearable measurement instruments to monitor biomechanical/physical exposures and assess associated risks, the instruments can also assist work technique training at the individual worker level. To do so, the information can be fed directly back to the user to instruct and guide the user to alter undesired movements or reinforce beneficial movements. Feedback training has several applications, including rehabilitation and sports (for an overview of the use in these domains, see, e.g., [70,71,72,73,74,75]) and for primary prevention of MSDs (i.e., preventing MSDs before they ever occur) in occupational settings [85]. When used in primary prevention, training should typically not be used isolated. To be effective, prevention strategies are recommended to comprise multiple measures targeting organizational, technical, and individual aspects [248], and should primarily follow the so-called hierarchy of controls [219]. According to the hierarchy of controls (Figure 3), hazards should be moved according to a preferred order of action based on their general effectiveness: elimination, substitution, engineering controls, administrative controls, and personal protective equipment (PPE).

#### 3.3.1. Work Technique Training

If the hazards cannot be eliminated or reduced to an acceptable level by elimination or substitution the hazard, or by implementing engineering controls (such as lifting equipment) – then, work technique training or training to increase the worker’s capacity should be used (i.e., strength training programs may serve as complementary strategies to reduce hazards [249,250]). Work technique training often comprises education on body mechanics and back care, training on lifting techniques, and how to handle mechanical lifting devices [250,251,252]. The practical training is, for convenience, often performed on a few simulated work tasks, as opposed to being performed in a real work context. This may pose a problem when transferring the achieved learning to more complex (real) situations [252,253]. The effectiveness of this traditional training has been questioned in recent systematic literature reviews, showing, in general, little or no clinically relevant effect [254,255,256]. As commented by Denis et al. [252], this lack of effectiveness of traditional training of work technique may be attributed to the quality of the training, which can hinder the possibility of inducing motor learning to retain behavioral changes effectively and can be used in real complex work situations. Hence, to increase the quality of work technique training, Denis et al. [252] proposed that training should be performed in more real (or real) work situations and allow training for longer periods. Additionally, as discussed previously (see Section 3.1.1), the use of wearable motion capture instruments and systems may facilitate the detection of changes in biomechanical exposure due to their ability to collect data over full or several workdays and with high accuracy and precision. While traditional training using feedback often requires the presence of a trained instructor [253], some of the wearable motion capture systems have integrated automated feedback systems [85,87,99,104,105,257], which provide feedback of varies modalities such as audio, visual, or vibrotactile feedback (see Table 12 for an overview of various feedback characteristics), or a combination of several multimodalities.

#### 3.3.2. Feedback-Based Work Technique Training Using Wearable Motion Capture Systems

The effectiveness of a specific feedback modality over another is related to the circumstances of the specific context of use [258]. While visuals can provide rich information on the effect of postural strategies [87], they can interfere with another stimulus with adverse effects on performance. Audio feedback has, on the other hand, been found to be effective for reducing trunk flexion in patient transfer training [99], but it can be masked in situations with ambient noise and might be perceived as annoying by others. In those situations, vibrotactile feedback may be an attractive option [85,104,105]. The feedback can be provided to draw attention to successful executions (positive feedback) or undesired executions (negative feedback). While the intention of the former is to reinforce movements/executions, an example of the latter is to give feedback when certain pre-determined postural thresholds are reached or exceed, such as a trunk inclination of 30° or more [85]. As shown in Table 7, feedback can be provided in real-time, e.g., as soon as certain postural thresholds are reached (i.e., concurrent feedback). An alternative is to provide feedback at fixed moments, such as after completing a task (i.e., terminal feedback) [74]. The feedback can be initiated automatically by the system or based on the request of the receiver. Extensive use of feedback can obstruct learning and implementation of new motor strategies by hindering the person from using their own intrinsic feedback system. To avoid this dependence on external feedback, it has been recommended that some initial training with the assistance of external feedback should be used to sequentially reduce the frequency of the feedback (i.e., fading feedback) [74,111]. By doing so, the person can, to a greater extent, rely on the intrinsic feedback system of the human locomotor system to adopt new motor strategies that can be retained without external feedback.

The results of the relatively few studies indicate that feedback, such as vibrotactile feedback, can render a clinically significant reduction in occupational exposure to manual handling in the short term. The long-term effects of feedback provided by wearable motion capture systems are largely uncertain, primarily due to the scarcity of studies, especially those that have evaluated this in a real working context and that have follow-up periods of several months [85].

**Table 12:** Feedback characteristics taxonomy, partly based on Sigrist et al. [258] and Ribeiro et al. [74].

Category	Sub-Category	Description
Modality	Audio	Feedback is provided using various audible sources, typically the tone of verbal messages [99].
	Visual	Feedback is typically provided using (wearable and non-wearable) screens or projectors [87] or by ocular viewing without augmenting devices.
	Vibrotactile	Feedback is typically provided using a vibration motor attached close to the targeted body segment, such as on the upper arm or trunk [85,104,105].
	Tactile	The feedback that typically can be sensed by touch by the application of forces and/or vibrations to the skin or similar.
	Haptic	Feedback is perceived by touch by the application of forces and/or vibrations or motions (kinesthetic perception).
Unimodality vs. multimodality		Feedback is provided using a combination of different feedback modalities (e.g., a combination of audio and vibrotactile feedback), which often can further enhance learning [258].
Positive/negative feedback (augment/reinforcement—suppress)	Negative	Sometimes referred to as posture-correction feedback or error amplification feedback. Typically provided to make the person aware of undesirable motor strategies to initiate alternative motor strategies to prevent or minimize their occurrence [85,104,105].
	Positive	Feedback is typically provided after or during a successful execution (or trial) to reinforce successful motor strategies and to enhance motivation.
Uni-channel versus multi-channel	-	This can refer to the number of locations where the feedback is given, including if different feedback transmitters are given simultaneously or in sequence (i.e., feedback is given at one location and, when it ends, is given at another location).
Timing	Concurrent	Feedback is provided simultaneously with task execution [85,104,105].
	Terminal	Feedback is provided after task execution [99].
	Fading	The frequency or duration of feedback is reduced with time.
Initiation	System determined	Feedback is initiated by the system, e.g., when reaching or exceeding pre-determined thresholds, such as posture angles or angular velocities [85].
	Self-controlled	Feedback is initiated at the request of the receiver [253].
Miscellaneous	-	An additional variation of the feedback characteristics can be related to the pitch and tonality of the feedback, as well as the duration, and if it is given as one or several signals at a certain temporal pattern. Other aspects refer to the intensity of the signal.

## 4. Discussion

As shown in this overview, motion capture instruments and systems have several applications for the prevention of WMSDs. The use of these instruments for monitoring movements and postures of the trunk and upper extremities has increased over the last two decades among researchers and OSH practitioners [46,83,259]. Currently, several challenges and opportunities exist for the prevention of WMSDs.

### 4.1. Challenges in Occupational Applications of Wearable Technology

Currently, several challenges exist for the broader use of these wearable motion capture instruments, such as the complexity of the equipment initiation and assessment, analyzing the data which are related to both the hardware used (e.g., accelerometers or IMUs), choice of filters, and software to support the data collection and analysis [82,83,116,260,261].

Technology readiness is naturally an important factor in the popularity and user acceptance of new technologies, which is also reported for wearable technology in sports [262]. It should be noted that technological readiness relates to the whole solution and not only the hardware, e.g., the sensing and processing part. Many solutions used in research, e.g., Xsens (Xsens Technologies B.V., Enschede, The Netherlands), WearNotch (Notch Interfaces Inc, New York, NY, USA), Axivity (Axivity Ltd., Newcastle, UK), Rokoko (Rokoko Electronics ApS, Copenhagen, Denmark), and LPMS-B2 and LPMOCAP (P-RESEARCH Inc., Tokyo, Japan) are mainly developed for motion capturing in sports, games, or rehabilitation. Hence, even if there are overlapping kinematics, such systems are not designed for ergonomics, and their proper use needs extra efforts for performing analysis and risk assessment, which might reduce usefulness and efficiency for regular use in risk assessment.

Wearables are becoming smaller and, over time, less costly. There are several surveys about hinders to the adoption of wearable devices in the workplace [263,264,265,266]. Existing risk assessment systems based on wearables are still in an early stage of maturity, and several factors need to be improved. In addition to the precision and technical functionality, some examples include high usability levels and critical factors for any system, including wearable-based ergonomic risk assessment and work technique training systems. Several factors influence feasibility and usability, including the running time, e.g., the battery life [263], the long-term wearer comfort (when intended to be used for extended periods) [85,267], the time needed for donning and doffing (including calibrating the system and cleaning to ensure hygiene), stability of wireless communication, e.g., Bluetooth or WiFi, and the logistics of replacing or charging the batteries. The usability of a wearable system also includes the analysis and reporting software. The usability should be ensured for different users, particularly OSH practitioners, researchers, and, eventually, workers. The relative importance of specific features can differ for different user populations [268,269]. For research purposes, the demands for precise and accurate measurements are often preferred even at the cost of more resources put into preparations (including calibration time and applications of the instruments), longer data collection periods, larger samples, and the complexity of the instruments and systems used. For OSH practitioners who often have more limited resources [41], there is often a higher demand for ease of use and shorter time for preparation, measurements, and analysis. The current relatively low use of wearable motion capture instruments (even among highly skilled experts from a high-income country) shown by Lowe et al. [46] calls for opportunities for increased efforts by developers with the aim of increasing the overall usability of future wearable motion capture devices.

A challenge in the everyday use of wearables at the workplace, especially in industrial settings, is due to heavy machinery and strong magnetic fields potentially interfering with wireless communications and disturbing sensors, such as magnetometers [116,185]. This makes the use cases that rely on real-time transfer data less reliable. Considering these scenarios is essential to ensure the solutions’ robustness in different environments.

Furthermore, it is essential to guarantee the worker’s privacy while collecting and handling their data [263,265]. The benefit of proper risk assessment and work technique training might not be very obvious for all workers and stakeholders, e.g., managers [270], which might hinder user acceptance.

Widespread use of wearable technologies in ergonomic risk assessment is not eliminating the need for OSH practitioners but could potentially steer some of the timely observations. Increased use of wearables has the potential to contribute to more efficient risk management by providing quantitative data on key performance indicators related to OSH. The accessibility of such OSH-related key performance indicators has been linked to the more efficient design of preventive measures [40] and the decreased likelihood of ergonomic issues being neglected [271], and has been found effective in facilitating decision-makers to take measures [272].

### 4.2. Validity Concerns and Risk Criteria

In the process of making the new wearables available to support the prevention of WMSDs on a large scale, and as mentioned above, optimal fusion algorithms should be identified and recommended. For measures that concern inclination/elevation from the vertical line, the gyroscopes and accelerometers of the IMUs facilitate very high validity when compared to a gold standard [273,274].

For the wrist, the angular velocity may today be computed from the gyroscope signals with adequate validity. When it comes to angles between two body segments, the magnetometer is needed for the wrist angle, where the movements may be in the horizontal plane. It is, however, more challenging to reach acceptable validity in wrist angle measurements in occupational settings using IMUs than in measurements of arm and trunk inclination, for which the magnetometer is not needed. In the technical data used to develop the action levels, as mentioned earlier, there was no clear association between wrist posture (measured with goniometers) and pain. Wrist posture is, however, an established risk factor for WMSDs, such as carpal tunnel syndrome [275], and more effort is needed to develop a robust and practical solution for accurate wrist posture measurements.

As discussed earlier, the angular velocity, which is primarily affected by sensor type, type of velocity (i.e., inclination or generalized velocity), and filter, can, at least in theory, be harmonized by applying conversion formulas [83]. Given that the existing conversion formulas only comprised two manual handling occupations/tasks, future studies are needed to validate those in other occupations and work tasks. Additionally, the overall acceleration during walking, and possibly other arm and trunk movement patterns, could influence the conversion. The generalizability of such conversion equations to multiple occupational groups is yet to be tested, and hence more research is needed.

Today, several observational tools include qualitatively based risk criteria that cannot easily be converted to objective quantitative metrics, such as repetitiveness. In the qualitatively-based risk criteria, repetitiveness is often quantified as the number of repeated movements during a time unit [276]. The former is difficult to measure technically without an amplitude criterion for how large angular change is required to be counted as a new movement. Mean power frequency (MPF) has been suggested as a measure of repetitiveness [277], but it excludes information on the amplitude of the movements. Instead, angular velocity, which includes both frequency and amplitude, can be used as a proxy. As seen in Table 3, angular velocity is included in many studies that use wearable motion capture instruments and can also be connected with quantitative threshold values, such as the action levels by Arvidsson et al. [58]. If we, internationally, also could agree on which risk criteria to use together with measured exposure (the action levels [58] could serve as a base), it would make the precise technical measurements more attractive among OSH practitioners (including professional ergonomists), such as the earlier comparison to noise and vibration exposure.

### 4.3. The Need for International Collaborations

Among the studies in Table 3 and Table 4, there is a large variation of methods used in addition to differences in hardware, the analysis of the collected signals, the location of where the sensors are placed, and the reference posture (i.e., the definition of the zero-angle posture of each body segment), which all influence the results. Based on this, future steps are needed to standardize the protocols, including data collection and analysis and facilitate the comparison and merging of data from different research groups. As previously mentioned, an example of such an initiative is the European collaborative project of the Partnership for European Research in Occupation Safety and Health (PEROSH). PEROSH includes 14 national OSH institutes [209]. Hence, to merge exposure data, the datasets should be very similar and have very similar metrics of health status (such as pain dimensions) collected using similar methodologies and instruments (often questionnaires or clinical examinations). One example of such a validated instrument is the Health Surveillance in Adverse Ergonomics Conditions protocol [278], which was used in the large dataset [279] and formed the basis of the action levels by Arvidsson et al. [58].

Another example of an international project to standardize the protocols of measurements, which was also performed by PEROSH [280], resulted in a common exchange platform form for measurements of occupational, physical activity, physical workload, and health data to facilitate the merging of multiple studies to identify risk criteria.

These examples of efforts (standardizing methods) are welcome, and the researcher and institutes participating in the projects will likely conform to those standards. However, a similar effort by a highly respected global Task Group would likely have a larger impact. Today, there are many open-source analysis algorithms. If such a global Task Group agreed upon standards, for e.g., the trunk, arms, head, and possibly wrists (including sensor type, placement of sensors, lowest sampling frequency, low-pass filter, reference posture and procedures, and even fusion algorithm), an open-source software package could support those standards.

### 4.4. Opportunities in Occupational Applications of Wearable Technology

A broader use can increase the general quality of risk assessments because of the higher accuracy and precision of exposure measurements relative to what can be obtained from self-reports and observational tools [47,113,239]. Increased use of motion capture instruments can contribute to more accurate exposure measurements with decreased misclassification of exposure profiles (i.e., high, medium, and low exposed groups or individuals) and greater possibilities to detect actual changes in exposure levels following implemented measures [238,239].

Contrary to observational tools that typically are applied on a shorter period intended to capture the exposure of a task or job, an advantage of the wearable motion capture instruments is the possibility for longer (continuous) measurement periods, which typically reduces the random error related to exposure variability (such as within and between workdays [113,174,281]). Other emerging opportunities are the possibilities for real-time exposure assessment (and risk assessment), which also can be fed back to the wearer to reduce adverse exposures by improving the work technique (altered postural behavior) without the need for a trained instructor [105]. Evidence of the long-term effectiveness of such feedback training is scarce and serves as an emerging research field [85].

Other emerging research fields of wearables include artificial intelligence (AI) and machine learning popularity of wearable devices, which ensure access to big data needed for AI applications. The state of the art of machine learning application in WMSD prevention has been reviewed by others [282] and is out of the scope of this review. However, a few contemporary applications of AI applications and machine learning related to motion capture that are relevant for exposure measurements and risk assessments can be mentioned:

The use of video-based motion-capturing systems using AI methods from computer vision as a base for risk assessment [283].

The use of AI for reducing the noise and artifacts from signals and analysis using wearable sensors and devices is reported. Examples of such AI use include sensor placement/assignment [284], removing motion artifacts, and compensating for the looseness of wearables [285].

Pattern recognition and predictive models allow for calculating risk scores using different risk assessment methods. Examples of such usage are reported for risk assessment of lifting action using RULA [112], the Revised NIOSH Lifting Equation (RNLE) [286,287], and accurate tracking of human activity recognition [288,289,290]. Pattern recognition can also be used to identify subject-specific kinematic fatigue responses, such as identifying early signs of fatigue in manual handling operations and training the machine learning to learn the subject’s normal movement patterns [291].

Recent advances in the development of generative AI models in other fields, especially in the field of natural language processing (NLP), are creating a lot of media hype, e.g., GPT-3 [292] and ChatGPT (OpenAI, San Francisco, California, USA). Generative Pre-trained Transformer (GPT) models are trained using large text datasets, allowing them to generate natural language responses that mimic human speech patterns. This makes them efficient in creating virtual assistants capable of providing targeted information for a broad range of applications. New applications may potentially be useful for guidance related to risk-reducing measures. SlimMe [293] is an example of a chatbot capable of supporting weight management. Given the short time since the release of ChatGPT, i.e., 30 November 2022, there should be more areas where this kind of technology may be useful, and there are already reports suggesting the potential for an AI-assisted medical education assistant [294]. We hypothesize that there is also a potential for developing specially trained GPT models that can provide tailored ergonomics evidence-based guidance for risk-reducing measures, such as work design and individual recommendations targeting work technique training (similar to a virtual instructor) based on the subject’s specific work postures and movements collected from the wearables.

Other emerging fields of research in the prevention of WMSDs are augmented reality (AR), virtual reality (VR), and similar computer-generated environments [295]. For an overview of these research fields, the reader is referred to other sources [295,296,297], but some aspects can be highlighted related to the prevention of WMSDs. Augmented reality, in which virtual representations of objects are overlaid onto real physical contexts [295], is used in some of the systems providing feedback to the wearer, as shown in Table 7. One such example is the vision-based system evaluated by Vignais et al. [87], in which the worker is represented as a manikin, and where adverse exposures are visualized as colored body parts of the manikin to indicate the severity of the hazardous exposure of each gross body segment. Virtual reality, on the other hand, typically provides an immersive virtual environment with real-time interactive graphics [295] and has multiple applications linked to the prevention of MSDs, including training of tasks (simulations), evaluating design choices of layout or components, and designing work tasks that can be performed to reduce adverse biomechanical exposures [298,299]. The use of wearable motion captures, such as IMUs, allows for the presentation of the user’s postures and movements in the virtual movements [298,300] and can also be integrated with some of the observational risk assessments presented in Section 3.2.2.

### 4.5. Review Limitations

The overview’s included studies were obtained using a multi-faceted approach that involved screening recent literature reviews and overviews, utilizing related articles and reference lists of the retrieved articles (i.e., “snowballing”) [80,81], and additional searches in Web of Science that applied specific inclusion criteria. Given the broad focus of the overview, we chose not to include all relevant studies but rather a selection that could best highlight current applications and discuss relevant challenges and opportunities. Due to this approach, we did not utilize well-established protocols, such as PRISMA [301] or AMSTAR [302], which may affect the reproducibility of this overview. Consequently, some relevant literature may have been overlooked, leading to limitations in interpreting the precise frequency of technology usage. However, the multiple-approach strategy enabled us to identify a fair number of studies compared to some recent systematic literature reviews, as indicated in Table 2.

## 5. Conclusions

The use of wearable motion capture instruments and systems in field applications in ergonomics is increasing. The wearable technology shows potential for broad applications in activities aimed at preventing work-related musculoskeletal disorders, including exposure assessment, risk assessment, and work technique training. Recent developments in artificial intelligence and machine learning also show potential for applications for data analysis and risk assessment related to biomechanical exposure.

While the miniaturization and improved usability and wearability can expand the use to more occupational settings and increase the use among occupational safety and health practitioners, several fundamental challenges remain to be resolved which, for some, may require more international collaborations and standardized protocols to enhance the potential of the technology. When compared with traditionally used observational assessment tools, the use of wearable technology adds several advantages in exposure measurement and risk assessment, including increased accuracy and precision of the measurements, the possibility for longer data collections, real-time exposure visualization, and automatic risk assessment comprising full workday exposures using a range of existing tools as inputs.

To what extent work technique training strategies using wearables is an effective intervention strategy to reduce WMSDs is still uncertain, as well as how the training protocol should be designed to be effective from a long-term perspective. Still, wearable technology enables the automatic integration of several existing observational tools, allowing for real-time feedback or external monitoring.

The future opportunities of increased usage of wearable motion capture devices for the prevention of work-related musculoskeletal disorders may require more international collaborations for creating common standards for measurements, analyses, and exposure metrics, which can be related to epidemiologically based risk categories for work-related musculoskeletal disorders.

## Figures and Tables

**Table 1:** Definitions of the terms related to wearable devices and wearable technology were selected by rank-order by the number of scientific citations in Google Scholar.

Ref.	Term	Definition
[59]	Wearable electronics	“Devices that can be worn or mated with human skin to continuously and closely monitor an individual’s activities without interrupting or limiting the user’s motions”
[60]	Wearable technology	“An application enabled computing device that accepts and processes inputs. This device is generally a fashion accessory usually worn or attached to the body. The device could work independently or be tethered to a smartphone allowing some kind of meaningful interaction with the user. The wearable product could be on the body (like a smart patch), around the body (like a wristwatch or a headband), or in the body (like an identification sensor embedded under the skin or a sensor attached to the heart monitoring cardiac aberrations)”
[61]	Wearable technology	“Sensors and/or software applications (apps) on smartphones and tablets that can collect health-related data remotely, i.e., outside of the healthcare provider’s office. The data can be collected passively or may require a user’s input.”
[62]	Wearable device/technology	“Small electronic and mobile devices, or computers with wireless communications capability that are incorporated into gadgets, accessories, or clothes, which can be worn on the human body, or even invasive versions such as micro-chips or smart tattoos”
[63]	Wearable device	“A tiny package with powerful sensing, processing, storage, and communications capabilities, and the term can refer to any electronic device or product designed to provide a specific service that can be worn by the user.”
[64]	Wearable device	“Any electronic device or product designed to provide a specific service that can be worn by the user. Wearable technologies are unique in their requirements and functions as the technology incorporate computer and electronic technologies to clothing and other accessories. Typical wearable devices may include components such as sensors, speech recognition technologies, positioning chips, displays, and special monitoring devices. They must be self-powered and fully functional in order to provide access to information anywhere and at any time.”
Our definition	Wearable device	Gadgets, accessories, or clothes with incorporated self-powered electronics and software that are capable of sensing, processing, and storing, and have communication capabilities that can be comfortably worn on the human body or be implanted on or under the skin, and that are not perceived as obtrusive and hindering performance (such as work performance).

**Table 2:** The sources where the 28 peer-reviewed articles on ambulatory motion capture systems were identified.

Identified Articles		Sources (Screened)				
		Systematic Literature Reviews				Original Article
Ref.	Year	Lee et al. [84]	Ranavolo et al. [76]	Stefana et al. [63]	McDevitt et al. [72]	Lind et al. [85]
[86]	2009	X	X	-	-	X
[87]	2013	X	X	-	-	X
[88] ^2^	2013	-	-	-	-	-
[89,90]	2014	X	-	-	-	-
[91]	2014	-	X	X	-	-
[92]	2014	X	-	-	-	X
[93]	2015	X	-	-	-	-
[94,95]	2016	X	X	X	-	-
[96] ^2^	2017	-	-	-	-	-
[97]	2017	-	X	X	-	-
[98]	2017	-	-	X	-	-
[99]	2018	X	-	-	-	X
[100] ^2^	2018	-	-	-	-	-
[101]	2018	X	-	-	-	-
[102,103]	2019	X	-	-	-	-
[104]	2020	X	-	-	-	X
[105,106]	2020	-	-	-	-	X
[107,108] ^2^	2020	-	-	-	-	-
[109]	2020	X	-	X	-	-
[110] ^2^	2021	-	-	-	-	-
[111]	2021	-	-	-	-	X
[112] ^2^	2022	-	-	-	-	-
[85] ^1^	2023	-	-	-	-	X

Notes: ^1^ published as “in-press” in 2022; ^2^ other identified studies from searches in Web of Science.

**Table 5:** The five action levels for postures and angular velocities of the arm and wrist were derived from Arvidsson et al. [58].

		10th Percentile	50th Percentile	90th Percentile
Angular velocity	Arm *	-	60°/s	-
	Wrist (non-forceful work)	-	20°/s	-
	Wrist (forceful work)	-	15°/s	-
Posture	Arm (non-supported)	-	30°	60°

Notes: * the arm angular velocity refers to generalized velocity [213] based on three-axial accelerometers used with a low-pass filter of 5 Hz [214].

**Table 6:** Examples of commonly used or recently developed observational tools targeting different types of work exposure and body segments.

Tool	Targeted Work Exposure	Targeted Body Segment
Strain Index [221]	repetitive manual handling	arm, hand/wrist
Revised Strain Index [222]	repetitive manual handling	arm, hand/wrist
Distal Upper Extremity Tool [223]	repetitive manual handling	arm, hand/wrist
ART [224]	repetitive manual handling	arm, hand/wrist, neck, trunk
HARM [225]	repetitive manual handling	arm, hand/wrist, neck,
Revised NIOSH Lifting Equation [211]	manual lifting/lowering	back, whole body
Lifting Fatigue Failure Tool [226]	manual lifting/lowering	back
RAMP II [51]	various manual handling tasks, e.g., repetitive manual handling, lifting/lowering, pushing/pulling, and demanding postures	whole body
RULA [227]	demanding postures and force exertion	whole body
REBA [228]	demanding postures and force exertion	whole body
OWAS [229]	demanding postures and force exertion	whole body

**Table 8:** A sampling of demanding postures targeting mainly single demanding events or cumulative exposure during worktime (i.e., percentage of the time of the task performed, or minutes/hours per workday) in a selection of observational risk assessment tools.

Tool	A sampling of Exposure to Postures	
	Single Demanding Events	Cumulative Exposure
RULA [227]	X	
REBA [228]	X	
OWAS [229]		x
ART [224]		x
HARM [225]		x
RAMP II [51]		x

**Table 9:** In a selection of observational risk assessment tools, upper arm flexion/extension angles are in the sagittal plane in relation to the line of gravity (or in relation to a neutral upper arm posture).

Tool	No. of Categories	Neutral Zone	Non-Neutral Zone (Forward)	Non-Neutral Zone (Backward)
RULA [227]	5	−20° ^1^ to 20°	20–45°, 45–90°, >90°	<−20° ^1^
HARM [225]	3	0° to 30° ^2^	>30° ^2^	<−0° ^1,3^

Notes: ^1^ negative values denoted backward flexion (i.e., extension); ^2^ according to Douwes et al. [243], while the threshold for the neutral zone is 20° according to Douwes et al. [225]; ^3^ only stated as “the upper arm is raised backward without arm support.”

**Table 10:** Neck flexion/extension (or head inclination) angles in relation to the line of gravity (or in relation to a neutral neck posture) in a selection of observational risk assessment tools.

Tool	No. of Categories	Neutral Zone	Non-Neutral Zone (Forward)	Non-Neutral Zone (Backward)
RULA [227]	4	0–10°	10–20°, >20°	−20° * (and more)
HARM [225]	3	0–20°	>20°	−10° * (and more)
RAMP II [51]	3	−9° to 29°	≥30°	−10° * (and more)

Notes: * negative values denoted backward flexion/inclination (i.e., neck extension).

**Table 11:** Time (and repetition) categories for the postures of the neck (head) in a selection of observational risk assessment tools.

Tool	No. of Categories	Forward Flexion	Extension (Backward Bending)
RULA [227]	2	posture is held static for a longer time than 1 min without a break, repeated more than 4 times per min	posture is held static for a longer time than 1 min without a break, repeated more than 4 times per min
HARM [225]	3	0–10%, 10–50%, >50% *	0–10%, 10–50%, >50% *
RAMP II [51]	5–7	<5 min, 5 min to <30 min, 30 min to <1 h, 1 h to <2 h, 2 h to <3 h, 3 h to < 4 h, >4 h	<5 min, 5 min to <30 min, 30 min to <1 h, 1 h to <2 h, >2 h

Notes: * the percentage refers to the percentage of the time the task was performed.

## Data Availability

Not applicable.
